# Self-Tuned Two-Stage Point Cloud Reconstruction Framework Combining TPDn and PU-Net

**DOI:** 10.3390/jimaging11110396

**Published:** 2025-11-06

**Authors:** Zhiping Ying, Dayuan Lv

**Affiliations:** 1 School of Mechanical Engineering, Zhejiang Sci-Tech University, Hangzhou 310018, China; 2023220503099@mails.zstu.edu.cn; 2 Zhejiang Key Laboratory of Intelligent Manufacturing Equipment for Flexible Functional Materials, Zhejiang Sci-Tech University, Hangzhou 310018, China; 3 Zhejiang Modern Textile Technology Innovation Center, Shaoxing 312000, China

**Keywords:** 3D reconstruction, geometric methods, self-tuned denoising, point cloud upsampling, PU-Net

## Abstract

This paper presents a self-tuned two-stage framework for point cloud reconstruction. A parameter-free denoising module (TPDn) automatically selects thresholds through polynomial model fitting to remove noise and outliers without manual tuning. The denoised cloud is then upsampled by PU-Net to recover fine-grained geometry. This synergy enhances structural consistency and demonstrates qualitative robustness under various noise conditions. Experiments on synthetic datasets and real industrial scans show that the proposed method improves geometric accuracy and uniformity while maintaining low computational cost. The framework is simple, efficient, and easily scalable to large-scale point clouds.

## 1. Introduction

A point cloud is composed of discrete 3D points and represents the standard output of 3D scanning. As a compact and efficient 3D representation, point clouds are widely used in reverse engineering, virtual reality, autonomous driving, robotics, and immersive telepresence [1]. However, the quality of raw point clouds is often degraded by sensor resolution limits and reconstruction errors, leading to significant noise, sparsity, and uneven distribution [2]. For example, LiDAR scanning of distant or small-scale objects frequently produces irregular, low-density noisy point sets [3]. Such issues severely affect the accuracy and robustness of downstream tasks, including reconstruction, rendering, and analysis. Therefore, effective denoising and upsampling of raw point clouds have become critical challenges in 3D vision.

To mitigate noise, numerous denoising strategies have been explored. Early approaches mainly relied on geometric modeling and optimization algorithms, such as moving least squares (MLS) surface fitting [4] and bilateral filtering [5], which reduced noise to some extent, but struggled to balance detail preservation and noise suppression. To improve robustness against outliers, robust statistical estimators were introduced, for example, sparse-representation-based normal estimation combined with graph regularization [6] or energy minimization. Although effective on simple geometries, these methods fail under heavy noise and require manual parameter tuning.

With the development of deep learning, data-driven point cloud denoising methods have gained popularity. Approaches such as PointCleanNet learn per-point displacement vectors for end-to-end denoising [7], but iterative processing may cause global shrinkage or detail loss. To reduce reliance on paired labels, unsupervised methods (e.g., TotalDn) leverage local density features [8], achieving promising results under low-noise conditions, but lacking robustness in complex scenes. In contrast, TPDn is a geometry-driven, parameter-free with self-tuned thresholds denoising approach that constructs a triangular mesh from the point cloud and extracts geometric features (e.g., normals and patch size). By fitting a polynomial distribution for automatic threshold selection, TPDn [9] achieves robust denoising without training or manual tuning, and demonstrates qualitative robustness on high-noise and complex-surface cases.

Despite significant progress in both denoising and upsampling, these two tasks have long been treated independently. Denoising methods effectively suppress noise, but cannot resolve geometric gaps caused by sparsity, whereas upsampling networks rely on clean inputs and may amplify noise or generate artifacts if applied directly to corrupted data [10]. This reveals the inherent coupling between noise and sparsity, which cannot be addressed effectively if the two problems are treated in isolation.

**Figure 1:**
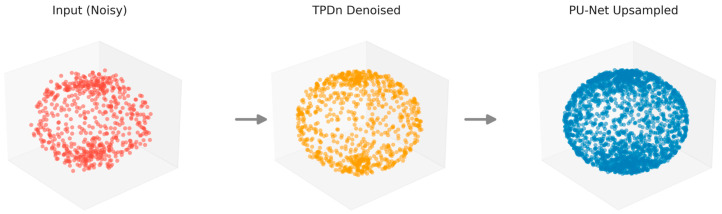
Illustration of the proposed two-stage pipeline on synthetic point cloud data. From **left** to **right**: The original noisy input, denoised results by TPDn, and the upsampled results by PU-Net.

To this end, we propose a complementary two-stage framework that combines a geometry-driven, parameter-free denoiser (TPDn) with a learning-based upsampling network (PU-Net) [11] to jointly handle noise and sparsity. Unlike a simple concatenation of two methods, our design emphasizes task-level synergy: TPDn not only improves the quality of PU-Net’s inputs, but also enhances its output uniformity and boundary fidelity, yielding superior overall performance compared to individual methods. As shown in Figure 1, the proposed two-stage pipeline progressively refines noisy point clouds through TPDn denoising followed by PU-Net upsampling.

The main contributions of this work are summarized as follows:

We introduce a synergy analysis between denoising and upsampling, revealing how TPDn’s adaptive threshold distribution statistically aligns with PU-Net’s feature manifold, thus improving learning stability and surface fidelity.

We construct a complementary two-stage framework that jointly addresses the coupled challenges of noise and sparsity.

We validate the effectiveness of the proposed method on ITODD [12], ModelNet40 [13], ShapeNet [14], and real-world industrial point clouds (collected in-house), demonstrating consistent improvements in accuracy, uniformity, and geometric fidelity over existing baselines [15].

A quantitative synergy analysis demonstrates that noise-feature suppression in TPDn directly enhances PU-Net’s uniformity and convergence stability, representing a principled coupling of geometric and learning-based reconstruction.

## 2. Related Work

### 2.1. Point Cloud Denoising

Point cloud denoising is a crucial step for improving data quality and enhancing the accuracy of subsequent reconstruction and analysis tasks. Traditional approaches typically rely on geometric priors, such as local surface fitting, smoothing, or statistical modeling, to remove noisy points that deviate from the underlying surface. For example, moving least squares (MLS) and polynomial fitting methods suppress noise [16] by fitting local patches and projecting points onto the estimated surface, but they often cause feature loss when handling complex geometries or high-noise scenarios. To address this limitation, robust statistical techniques such as RIMLS and WLOP [17], as well as optimization methods based on sparse representation and graph regularization [18], have been proposed. While these methods improve edge preservation, they generally require manual parameter tuning and exhibit limited adaptability across different datasets.

With the advancement of deep learning, researchers have increasingly adopted neural networks for denoising point clouds. PointCleanNet, for instance, predicts displacement vectors [19] for each point to achieve end-to-end denoising, but iterative refinement may cause global shrinkage. Unsupervised approaches [20] such as TotalDn employ neighborhood reconstruction losses to eliminate the dependency on paired training data, yet they often lack robustness in complex structures. Graph convolutional networks (GCNs) [21] have also been introduced to model local geometric relationships. More recently, Transformer- and diffusion-based point cloud enhancement methods [22] have shown potential for capturing global dependencies and generating plausible distributions [23]. However, these approaches typically require large-scale training datasets and incur heavy computational costs, which limit their generalization to noisy real-world point clouds.

In addition to learning-based strategies, geometry-driven parameter-free adaptive-threshold methods have also been explored. For example, TPDn constructs a triangular mesh and extracts geometric features to achieve robust denoising without training or manual parameter tuning. Such methods exhibit superior generalization and efficiency under high-noise and complex-surface conditions. Compared with deep learning models, geometry-driven approaches offer stronger cross-scene adaptability and can also serve as preprocessing modules that complement subsequent upsampling networks.

### 2.2. Point Cloud Upsampling

Point cloud upsampling, also known as point cloud densification, aims to expand a sparse point set into a denser, more uniform, and surface-faithful representation. Traditional approaches are largely based on geometric optimization strategies. For example, the Weighted Locally Optimal Projection (WLOP) [24] method formulates an energy function that projects points onto locally optimal surfaces while introducing repulsive forces between points to maintain uniform distribution. Subsequent improvements have incorporated density control and edge-awareness mechanisms, which enhance boundary preservation and local filling quality to some extent. However, these methods are limited when dealing with sharp edges, non-smooth surfaces, or noisy inputs, and they heavily depend on accurate normal estimation and manual parameter adjustment.

### 2.3. Summary and Comparison with Prior Coupled Frameworks

Although several prior studies have attempted to couple denoising and upsampling within a unified or sequential framework, these approaches still face critical limitations. Traditional joint filters [16,17] often rely on manually tuned parameters and lack adaptability across different noise levels, making them fragile under real industrial noise.

Learning-based coupled models, such as Pointfilter [18] or encoder–decoder frameworks that simultaneously predict clean and dense point sets, tend to blur fine structures and amplify artifacts when trained on limited noise distributions. Moreover, most existing combinations are heuristic—they cascade two networks or optimization modules without theoretical coordination, which may cause information inconsistency between the denoising and reconstruction stages.

**Figure 2:**
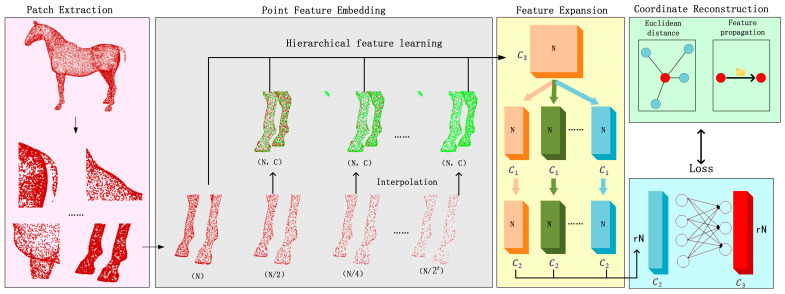
Overall flowchart of the two-stage reconstruction framework. Stage I: Structural optimization and denoising of noisy point clouds. Stage II: Density enhancement via upsampling. The staged design balances structural recovery and detail completion. Arrows indicate the data flow between modules, and different colors represent feature stages and point representations.

**Figure 3:**
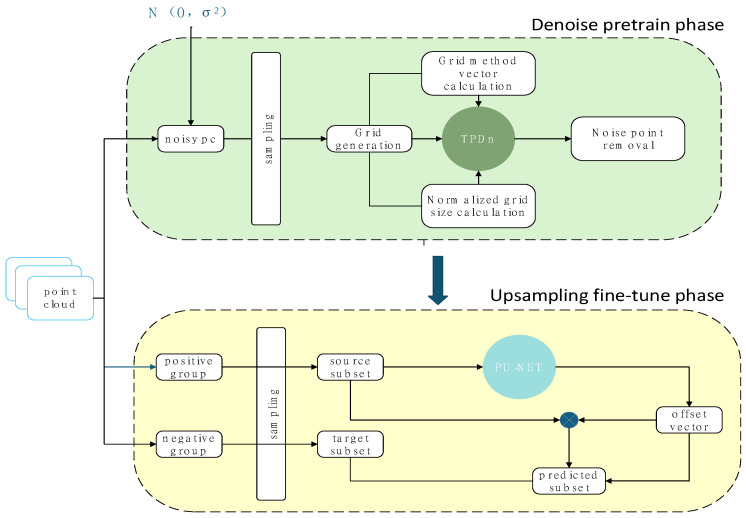
Module-level diagram. Stage I (TPDn) is training-free; Stage II (PU-Net) is learned. Loss terms apply only during Stage-II training; inference is TPDn → PU-Net.

In contrast, our proposed TPDn + PU-Net framework introduces a statistically grounded synergy: the adaptive thresholds in TPDn reshape the feature distribution of the inputs, leading to more stable and uniform learning in PU-Net. Meanwhile, deep learning methods leverage data-driven priors to achieve stronger generalization. PU-Net was the first deep neural network designed for point cloud upsampling [11]. It adopts a PointNet++ backbone to extract local geometric features, followed by multi-branch feature replication and MLP-based decoding to generate expanded point sets. PU-Net employs a joint loss function combining Earth Mover’s Distance (EMD) and repulsion loss, ensuring that the generated points are both surface-faithful and uniformly distributed. Figure 2 shows the training/validation/inference timeline (Stage-I → Stage-II). In contrast, Figure 3 details the module-level architecture (TPDn features and thresholding; PU-Net backbone → feature expansion → MLP heads).

Building upon PU-Net, a series of improved methods have been proposed: MPU introduces progressive multi-stage upsampling [25] for higher-quality expansion; EC-Net enhances edge preservation [26]; PU-GAN incorporates generative adversarial learning [27] with a discriminator to improve realism and distribution plausibility; PU-GCN leverages graph neural networks [28] to propagate features and refine local detail modeling; and Meta-PU adopts meta-learning [29] to generalize across different upsampling ratios and shape categories. In addition, unsupervised and self-supervised approaches [30] have been explored to recover missing points from incomplete data.

Overall, point cloud upsampling techniques have evolved from traditional optimization schemes to deep learning paradigms [31]. In this work, we select PU-Net as the upsampling module due to its simplicity, strong baseline performance, and broad adoption. Combined with the preceding TPDn denoising stage, PU-Net receives high-quality inputs, enabling the generation of dense point clouds that better preserve geometric structures and surface details.

## 3. Methodology

We propose a two-stage framework for point cloud reconstruction that sequentially performs denoising and upsampling, as illustrated in Figure 3. In the first stage, the point cloud denoising algorithm (TPDn) processes noisy inputs to remove outliers and noise perturbations while preserving the underlying geometric structures. In the second stage, the point cloud upsampling network (PU-Net) takes the denoised data and generates higher-density point sets, producing more detailed representations of object surfaces. By decomposing the task into two submodules, each focusing on its specific objective, the proposed framework collaboratively transforms raw scan data into high-quality dense point clouds. The following subsections introduce each component and the overall system design, where training and loss functions are discussed only for the upsampling network. This interaction forms a cross-stage feedback where the denoising thresholds statistically regulate PU-Net’s receptive-field variance, ensuring robustness under heavy noise.

TPDn is a parameter-free adaptive denoising method that leverages geometric features and mesh-based processing to accurately identify and remove noise. Unlike conventional deep learning models or iterative optimization techniques, TPDn distinguishes noisy points from valid structures by constructing a triangular mesh and extracting geometric descriptors. It adopts a global–local feature fusion strategy on the mesh, enabling fine-grained denoising while maintaining the global shape of the point cloud. This makes TPDn particularly effective for denoising point clouds acquired in complex environments.

In the initial stage of TPDn, the raw point cloud is converted into a mesh structure using the Greedy Projection Triangulation algorithm [32], resulting in a set of triangular patches. Mesh construction not only captures local geometric structures with high fidelity, but also establishes spatial relationships between points through patch connectivity, thereby enhancing local feature extraction during the denoising process. In particular, TPDn extracts mesh normals and normalized patch sizes, which serve as reliable indicators for distinguishing noise from valid points.

The computation of mesh normals reflects variations in local curvature, which is crucial for detecting noisy points. Suppose $f_{i}$ and $f_{j}$ are the normals of two adjacent triangular patches. The angular deviation between the normals can be measured as:

(1) $$\theta_{\mathrm{ij}}=\arccos(\frac{f_{i}\times f_{j}}{∥f_{i}∥∥f_{j}∥})$$

This measure captures the continuity of normal variation in a local region. Ideally, noisy points induce abrupt changes in normal direction, which can be distinguished through such feature variations. In addition, the normalized patch size is employed to quantify the distribution and consistency of triangular patches. During denoising, abnormal changes in patch size typically indicate noise, since noisy points lead to unnatural expansions among neighboring vertices. The normalized patch size is defined as the area of the triangular patch formed by the neighboring vertices, expressed as:

(2) $$S_{i}=\frac{1}{2}∥\left(b_{i}-a_{i}\right)\times \left(c_{i}-a_{i}\right)∥$$

where $a_{i},b_{i},c_{i}$ are the vertices of the patch and ||.||denotes the vector norm. This definition ensures dimensional consistency and accurately reflects the local geometric scale of the patch. TPDn employs a statistically optimized, training-independent thresholding strategy to determine the boundary between noise and non-noise points through a global optimization scheme, thereby avoiding the complexity of manual threshold selection through automatic, data-driven thresholding. Specifically, the method uses high-order polynomial fitting of feature distributions to automatically select noise thresholds. Assuming the fitted function is expressed as:

(3) $$f\left(x\right)=a_{n}x^{n}+a_{n-1}x^{n-1}+\cdots +a_{1}x+a_{0}$$

where x denotes feature values and $a_{n},a_{n-1},\cdots ,a_{0}$ are polynomial coefficients. By analyzing the second derivative f′′(x), the inflection point of the distribution can be precisely located, which corresponds to the boundary between noisy and valid points. This enables automatic threshold determination without manual intervention. The advantage of polynomial fitting lies in its ability to capture subtle variations in point cloud features and adapt the threshold to different noise levels.

To evaluate the robustness of the adaptive polynomial approximation, we conducted a sensitivity analysis with respect to both the polynomial degree (*n*) and the input noise level (σ). As summarized in Table 1, the denoising accuracy, measured by Chamfer Distance (CD) and Point-to-Face (P2F), remains stable when varying *n* = 4–6 under noise levels σ = 0.01–0.05. The variation across settings is within ±4%, indicating that the proposed polynomial thresholding scheme is robust to both polynomial order and noise intensity.

In practice, the polynomial degree n is empirically set between 4 and 6. Lower degrees tend to underfit feature distributions and blur the transition between noise and valid points, whereas excessively high degrees cause numerical oscillation and over-sensitivity to outliers. To ensure stability, the optimal degree is automatically selected by minimizing the Akaike Information Criterion (AIC) on the histogram of geometric features, yielding consistent thresholds across different datasets and noise intensities. This fitting process is computationally lightweight: it typically converges within 0.02 s for 10^3^ sampled features on a standard CPU, accounting for less than 5% of the total runtime of TPDn.

During the denoising process, several geometric metrics are used to evaluate denoising performance, including the Chamfer Distance (CD) [33] and Point-to-Face distance (P2F) [34], defined as:

(4) $$\mathrm{CD}\left(P_{C1},P_{C2}\right)=\frac{1}{N_{1}}\sum_{p_{1}\in P_{C1}}\underset{p_{2}\in P_{C2}}{\min}∥p_{1}-p_{2}{∥}^{2}+\frac{1}{N_{2}}\sum_{p_{2}\in P_{C2}}\underset{p_{1}\in P_{C1}}{\min}∥p_{2}-p_{1}{∥}^{2}$$

(5) $$P2F\left(P,M\right)=\frac{1}{\left|P\right|}\sum_{p\in P}\underset{f\in M}{\min}d\left(p,f\right)$$

where $P_{C1}$ and $P_{C2}$ denote the denoised and ground-truth point clouds, respectively, and $N_{1}$,$N_{2}$ are the number of points in each set. The Chamfer Distance ensures that denoised outputs remain close to the ground truth distribution.

In addition, TPDn incorporates an adaptive resampling strategy to ensure that the retained points remain representative. This strategy avoids discarding critical structural points while removing noise, thus maintaining robustness under complex environments and varying noise levels.

Specifically, adaptive resampling evaluates the local geometric saliency of each point based on normal variation and patch-size deviation. Points within regions of high curvature or edge continuity are assigned higher retention weights, while those with flat-surface redundancy or abnormal feature variance are more likely to be discarded. A soft threshold is applied to the normalized saliency map to determine the final mask. On average, approximately 8–12% of points are removed under low-noise conditions and up to 20–25% under high-noise cases, which balances denoising strength and structural completeness.

In summary, TPDn provides a parameter-free adaptive-thresholding solution for point cloud denoising. By optimizing geometric features and employing polynomial-based automatic thresholding, it eliminates the need for manual parameter tuning and iterative refinement. As a result, TPDn effectively removes noise while preserving the intrinsic structural features of point clouds, making it particularly suitable for applications such as robotics and autonomous driving, where high-quality point data are required.

**Figure 4:**
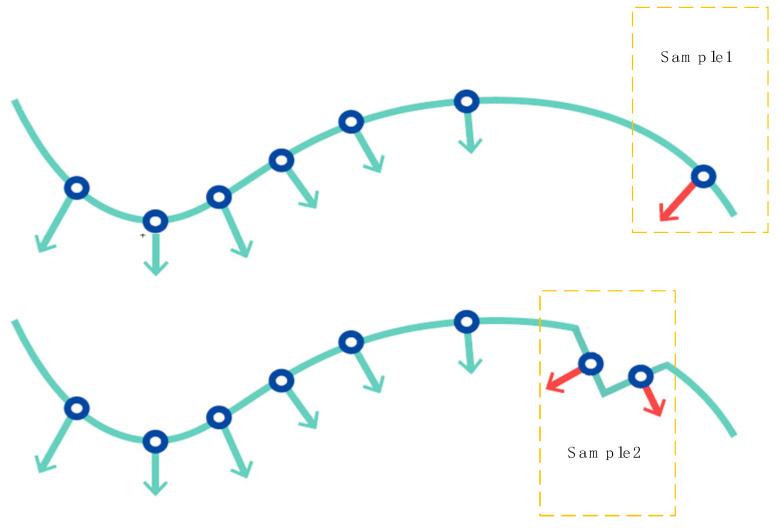
Illustration of two representative noise cases addressed by TPDn: Sample-1—dispersed noise with vertices far apart; Sample-2—local noise on surfaces with abrupt curvature changes. Red arrows indicate the direction of noise perturbations, and different colors represent separate sample cases.

Noise often interferes with the overall structure of point clouds. In general, outliers located far from the main surface are relatively easy to detect, whereas abnormal points close to the surface are more challenging to identify. In this work, we abstract such scenarios into two representative noise cases, as illustrated in Figure 4. sample-1 corresponds to dispersed noise where vertices are located far apart from each other, while sample-2 represents local noise on surfaces with abrupt curvature changes.

In the second stage, the denoised point set $P_{clean}$ is used as input and upsampled into a denser point set $P_{dense}$. In this work, we adopt PU-Net as the upsampling network, since it effectively reconstructs local surface details by processing overlapping patches. Specifically, for an input point cloud $P_{clean}$ containing N points, we first divide it into overlapping patches, each containing $N_{p}$ points. Patch generation is achieved by sampling seed points and grouping their k-nearest neighbors, similar to the PointNet++ strategy. Each patch is then processed independently.

Within PU-Net, each patch first passes through a feature extraction module, essentially a simplified PointNet++ backbone. By applying multilayer perceptrons (MLPs) and pooling operations, hierarchical features (e.g., 128 or 256 dimensions) are learned to capture curvature, local shape, and other geometric properties. Subsequently, the feature expansion module replicates each feature vector r times (where r is the upsampling ratio, such as r = 4), with multiple MLP branches introducing slight perturbations to generate diversified features. Intuitively, if a patch lies on a surface manifold, each branch produces slightly different position features. The expanded features (with dimensions $r\times N_{p}$) are finally fed into a coordinate reconstruction module (MLP) to generate the XYZ coordinates of the upsampled points. Each patch ultimately outputs $rN_{p}$ points. After merging and deduplicating overlapping patches, a dense point set $P_{dense}$ is obtained. Since the input $P_{clean}$ is largely noise-free, PU-Net can focus exclusively on interpolating new points along the surface without being disrupted by outliers.

During training, PU-Net employs the original joint loss design, consisting of the reconstruction loss $L_{EMD}$ and the repulsion loss $L_{rep}$. The reconstruction loss adopts the Earth Mover’s Distance (EMD) [35], which computes the optimal point-wise matching between the network output and the ground-truth dense point cloud, thereby ensuring distribution-level consistency. Compared with Chamfer Distance, EMD provides stricter optimization by enforcing overall distribution alignment. The repulsion loss $L_{rep}$ penalizes pairs of output points that are excessively close to each other, preventing point collapse and encouraging uniform spatial distribution. The overall upsampling loss is defined as:

(6) $$L_{upsample}=L_{EMD}+\alpha L_{rep}$$

where α is a weighting factor, and additional regularization terms may be included during training if necessary. In this framework, PU-Net is applied to noise-free inputs, enabling it to generate dense point clouds with high fidelity and uniformity.

The proposed framework adopts a decoupled pipeline to separately perform denoising and upsampling, simplifying the overall design and improving flexibility. First, the TPDn denoising method is validated on synthetic noisy point cloud datasets with clean ground-truth labels. Evaluation metrics include the aforementioned geometric measures such as Chamfer Distance and Point-to-Face (P2F) distance. In implementation, multiple samples are processed in batches (≈8 per batch), and results are averaged over three independent seeds (mean ± std). This denoising process requires no parameter training and relies solely on geometric features with automatic threshold selection.

After validating the denoising stage, we further train the PU-Net upsampling module. The training data pairs are constructed in the same manner as the original PU-Net paper: clean point clouds are randomly downsampled or sparsified to serve as inputs, while the original dense point clouds provide supervision labels. In this work, an upsampling ratio of r = 4 is adopted as the default configuration. At the patch level, PU-Net is trained with a joint loss function consisting of Earth Mover’s Distance (EMD) and repulsion loss. During training, patches are randomly sampled from the dataset, downsampled to 256 points as inputs, and the complete patches serve as labels. The network is trained for approximately 100 epochs using the Adam optimizer with an initial learning rate of 0.001 that decays progressively, until convergence on the validation set.

Once training is complete, the two modules are integrated for inference. For a new sparse and noisy input (e.g., depth camera/LiDAR scan data), TPDn is first applied to produce a purified point set, which is then fed into PU-Net for upsampling. The entire inference pipeline involves only forward computation and can process approximately 10,000-point data within a few hundred milliseconds on a modern GPU. Notably, although an end-to-end learning framework could theoretically replace TPDn, experimental results demonstrate that the decoupled strategy already achieves competitive performance while providing higher flexibility. For example, PU-Net can be replaced with PU-GAN or PU-Transformer as long as the input–output interfaces remain consistent.

**Figure 5:**
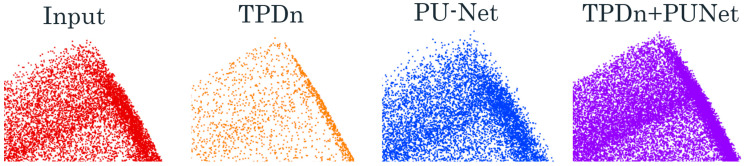
Denoising results on industrial part point clouds using different methods. Other methods tend to disrupt structural integrity or distort shapes, whereas the proposed TPDn provides more stable results.

By combining the geometry-driven denoising capability of TPDn with the learning-based upsampling ability of PU-Net, the proposed method produces dense and uniformly distributed point clouds while ensuring high input quality. Figure 5 illustrates this pipeline: TPDn removes noise and purifies the input, and PU-Net generates a denser reconstruction from the cleaned data. The following section introduces the experimental setup used to evaluate our approach.

## 4. Experimental Setup

To validate the effectiveness of the proposed two-stage framework (TPDn + PU-Net) for point cloud reconstruction, we conducted experiments on both synthetic and real scanned data and compared the results against several baseline methods. This section describes the datasets, noise simulation, training configurations, and evaluation metrics.

### 4.1. Datasets

Our experiments are based on publicly available 3D point cloud datasets. For synthetic data, we employ the MVTec ITODD dataset, which contains 120 training models and 27 testing models, covering diverse industrial objects such as screws, hubs, and bushings with high-quality point clouds. In addition, selected models from ModelNet40 [13] and ShapeNet [14] are included to increase shape diversity, where surface sampling is applied to obtain point sets.

### 4.2. Noise Simulation

To generate noisy inputs, two samples of corruption are applied to clean point clouds:

- Gaussian noise: Zero-mean Gaussian perturbations are added to each coordinate, with the standard deviation set to 0.5% or 1% of the object’s diagonal length, corresponding to low- and medium-level noise.
- Outlier noise: Approximately 5% of the points are randomly replaced or added outside the surface, simulating scanning artifacts.

For each model, two noise conditions are considered: low noise (Gaussian noise only, without outliers) and high noise (Gaussian noise plus 5% outliers).

During the PU-Net training phase, sparse point clouds are used as inputs. Specifically, sparse inputs are generated by randomly downsampling clean point clouds, while the original dense point clouds serve as supervision labels. For example, in PU-Net training, each patch is typically downsampled to one-quarter of its original size and used for 4× upsampling. During testing, both noise and sparsity are considered: input point clouds are first denoised by TPDn (whose adaptive resampling also alleviates sparsity to some extent), and the denoised results are then fed into PU-Net for upsampling. While our main quantitative evaluation focuses on the low-noise setting, quantitative metrics under high-noise conditions are also reported for reference, accompanied by qualitative visual analysis.

### 4.3. TPDn Configuration

As a geometry-driven method, TPDn requires no training. Each run processes approximately eight samples, and all metrics are computed over three independent random seeds to ensure statistical robustness. Additional rotations and perturbations are applied to simulate acquisition variations. The denoising performance is measured using Chamfer Distance (CD) and Point-to-Face (P2F) metrics.

### 4.4. PU-Net Training Configuration

PU-Net is trained on clean data. For each shape, 8192 points are sampled as ground truth, and a random subset of 2048 points is used as input to achieve 4× upsampling. The input point clouds are further divided into patches to enhance local diversity. Training is performed using the Adam optimizer with an initial learning rate of 0.001 that decays progressively, and the model converges after approximately 100 epochs.

### 4.5. Baselines and Comparative Methods

To highlight the contribution of the denoising stage, we include an additional baseline where PU-Net is trained directly on noisy inputs without any preprocessing. The results show that this approach performs significantly worse than the proposed two-stage framework, indicating that a dedicated denoising step is indispensable. A complete list of comparative pipelines is provided in 4.8, and the parameter configurations of TPDn and PU-Net are summarized in Table 2.

### 4.6. Evaluation Metrics

1. Chamfer Distance (CD): Measures the coverage of reconstructed point sets relative to the ground truth.
2. Earth Mover’s Distance (EMD): Evaluates global matching quality through optimal point-to-point correspondence.
3. Variance of 1-nearest-neighbor (1-NN) distances (V-NND): Assesses the uniformity of point distributions, with lower variance indicating better uniformity.
4. Point-to-Face Distance (P2F): When ground-truth meshes are available, computes both mean error and the 95th percentile error to quantify geometric fidelity.

In addition, qualitative evaluations are conducted using 3D visualizations to intuitively demonstrate noise removal and structural preservation.

### 4.7. Experimental Environment

All experiments are conducted on an NVIDIA RTX 4090 GPU, with PyTorch 2.0 and CUDA 11.8 as the software environment.

### 4.8. Comparative Methods

We evaluate the proposed approach against several baselines on the test datasets:

- TPDn only: Applies denoising directly to noisy and sparse inputs without upsampling.
- PU-Net only: Applies upsampling directly to noisy and sparse inputs without denoising.
- TPDn + PU-Net (ours): The proposed complete two-stage framework.
- PCNet + PU-Net: A two-stage pipeline where PointCleanNet (PCNet) replaces TPDn as the denoiser, for comparison against our approach.
- SOR + 3D-CNN (ours, baseline): A self-constructed baseline that combines the standard Statistical Outlier Removal (SOR) filter implemented in the Point Cloud Library (PCL) [36] with a 3D convolutional neural network (3D-CNN) upsampling module [37]. This combination is not taken from any specific prior work, but was designed for comparison purposes to evaluate the effect of classical geometric filtering followed by learning-based upsampling.

For fair comparison, all methods are trained on the same train/validation splits and evaluated on the same held-out test sets. Although advanced upsampling networks such as PU-GAN [27], PU-GCN [28], and Transformer-based models [22,23] have demonstrated strong performance, they target different objectives—mainly generative realism or large-scale category generalization—while our focus is to analyze the synergy between geometry-driven denoising and learning-based upsampling. To maintain a clear experimental scope and ensure reproducibility, this study adopts PU-Net as a representative and widely established baseline, allowing for a fair and interpretable comparison of the proposed two-stage framework. Since the framework is modular, more advanced upsamplers can be seamlessly substituted into Stage II without retraining the denoiser, and the same synergy principle would remain applicable. A brief comparison of representative upsamplers in terms of supervision type, computational complexity, and interpretability is summarized in Table 3. Future work will extend the proposed pipeline to PU-GAN, PU-GCN, and Transformer-based architectures to further evaluate the generalizability and scalability of the synergy.

Future work will incorporate quantitative comparisons with PU-GAN, PU-GCN and Transformer-based methods to further validate scalability and generalization.

Therefore, PU-Net was intentionally selected as a strong and widely adopted baseline, providing a clear and interpretable reference.

**Figure 6:**
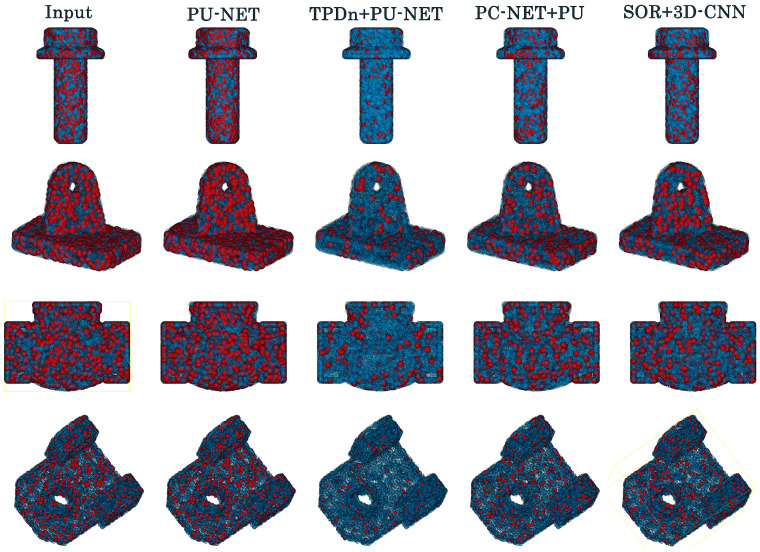
Comparison of reconstruction performance across different pipelines (PU-Net, TPDn + PU-Net, PCNet + PU-Net, and SOR + 3D-CNN) on multiple industrial part point clouds. Each row shows different object samples (e.g., cubic, hollow, or curved models), with error maps illustrating noise (red) and valid structures (blue).

Since our framework is modular, more advanced upsamplers can be easily substituted in Stage II without retraining the denoiser, and the same synergy principle would remain applicable. Representative results are illustrated in Figure 6. All methods share identical patching/triangulation parameters and the same noise model; PCNet is trained under the identical noise distribution.

To ensure fair quantitative comparison, all reconstructed point clouds were resampled to the same number of points (8192) before computing the Chamfer Distance (CD), Earth Mover’s Distance (EMD), and Point-to-Face (P2F) metrics. This normalization prevents artificially low error values caused by higher point densities and ensures that the evaluation reflects true geometric fidelity rather than point count differences.

Although advanced upsampling methods such as PU-GAN, PU-GCN, and Transformer-based architectures have achieved strong performance, they require complex adversarial training or large-scale datasets. In contrast, PU-Net was selected as the representative baseline for its lightweight design and reproducibility, which better aligns with the focus of this study on evaluating the synergy between denoising and upsampling rather than network complexity.

For reproducibility, Table 4 lists the main configuration parameters and references of all comparative methods. Default settings from the original papers or official repositories were adopted unless otherwise specified.

### 4.9. Configuration Efficiency Analysis

To demonstrate the automation advantage of the self-tuned TPDn design, we compare the number of trainable parameters, manual configuration requirements, and setup time with conventional denoising approaches. As summarized in Table 5, TPDn is entirely parameter-free and training-free, requiring no manual threshold adjustment or model pretraining. The configuration time is reduced by more than 60% compared with learning-based denoisers such as PCNet and TotalDn. This lightweight configuration significantly simplifies deployment while maintaining comparable accuracy, highlighting the self-tuned property of TPDn.

## 5. Results and Discussion

### 5.1. Quantitative Comparison

**Figure 7:**
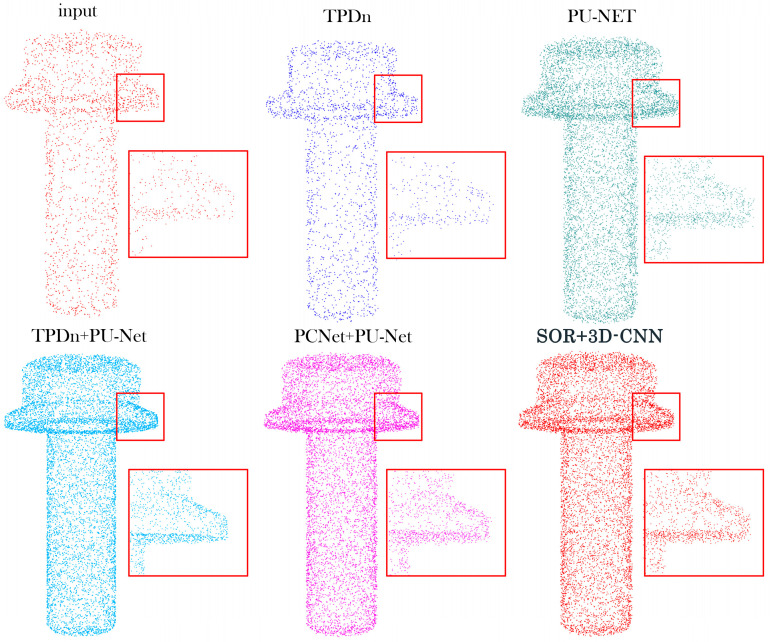
Reconstruction results under low-noise conditions (σ = 0.01, no outliers). Red boxes highlight the regions magnified for detailed comparison.

Under low-noise conditions, the input point clouds contain only slight Gaussian perturbations while largely preserving the overall structure (Figure 7). In this scenario, PU-Net alone can achieve reasonably acceptable reconstruction results (CD = 0.0041, EMD = 0.043, as shown in Table 6). However, our proposed two-stage framework (TPDn + PU-Net) still demonstrates superior performance, with CD and EMD reduced to 0.0018 and 0.013, respectively. This indicates that even in low-noise settings, the denoising stage significantly improves the quality of upsampling, validating the necessity of the design.

In terms of the mean point-to-face distance (P2F Mean), TPDn + PU-Net achieves the smallest error (0.012), outperforming PU-Net (0.038) and PCNet + PU-Net (0.016). This confirms that the output of our method more faithfully aligns with the underlying object surface. Regarding distribution uniformity, TPDn + PU-Net attains a nearest-neighbor distance variance of 0.0017, which is lower than PU-Net (0.0039) and PCNet + PU-Net (0.0025). This further demonstrates that the denoising stage helps improve the spatial consistency of the upsampled points.

By contrast, SOR + 3D-CNN achieves a Chamfer Distance of 0.0035 and a P2F error of 0.027, slightly better than PU-Net (0.0041/0.038). However, visual inspection reveals that it tends to remove valid boundary points along with noise, resulting in structural distortions and less faithful geometry overall. This suggests that traditional filtering-based methods still struggle to balance structural preservation and error control.

Overall, TPDn + PU-Net outperforms other comparison methods In terms of efficiency, the entire pipeline processes 10k-point data within 0.3 s on an RTX 4090 GPU and scales linearly (O(N)) with point count.

Batch-based subdivision enables handling of million-point scans with stable memory usage (<1.6 GB), demonstrating good scalability for industrial applications across three critical aspects: accuracy, uniformity, and geometric fidelity.

**Figure 8:**
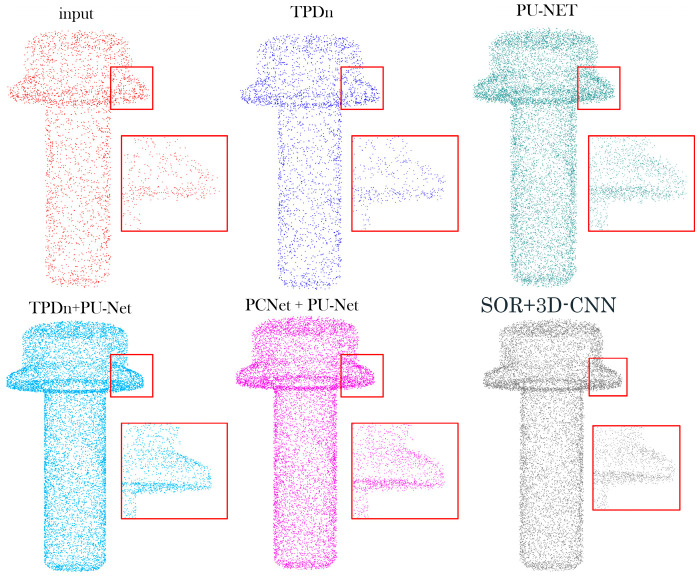
Reconstruction results under high-noise conditions (σ = 0.05 with 5% outliers). Red boxes highlight the regions magnified for detailed comparison.

To further evaluate the robustness of the proposed framework under severe noise, additional comparisons are presented in Figure 8 and Table 7. Figure 8 illustrates reconstruction results under a high-noise condition (σ = 0.05 with 5% outliers). The proposed TPDn + PU-Net effectively suppresses scattered outliers and preserves fine surface details, while the other baselines exhibit oversmoothing or residual noise. The quantitative metrics in Table 7 further confirm the robustness of our method, showing consistent improvements across all evaluation criteria.

**Figure 9:**
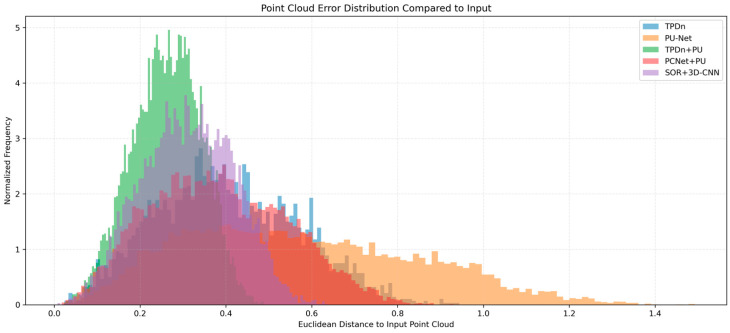
Normalized histograms of point-to-ground-truth distance (x-axis); y-axis shows normalized frequency.

Figure 9 illustrates the error distributions of different methods in the point cloud reconstruction task. The normalized histograms are generated by computing the Euclidean distance between each reconstructed point and its corresponding reference point. As shown, the proposed TPDn + PU-Net (green) achieves the most concentrated error distribution with a clear leftward shift, indicating higher geometric accuracy after denoising and upsampling. PCNet + PU-Net (pink) produces results close to our method, but remains slightly inferior. SOR + 3D-CNN (purple), although capable of generating dense outputs, exhibits prominent error peaks, suggesting limited capability when applied to noisy inputs without prior denoising. TPDn alone (blue) demonstrates a significant reduction in errors, confirming its effectiveness as a denoising module. By contrast, PU-Net alone (orange) yields the widest error distribution with a right-shifted peak, reflecting poor reconstruction quality.

These results confirm that the joint use of TPDn and PU-Net improves geometric accuracy and uniformity, outperforming the standalone denoising or upsampling models. This experiment validates the feasibility and advantage of the proposed two-stage framework in handling complex point cloud scenarios.

### 5.2. Poisson Surface Reconstruction Results

**Figure 10:**
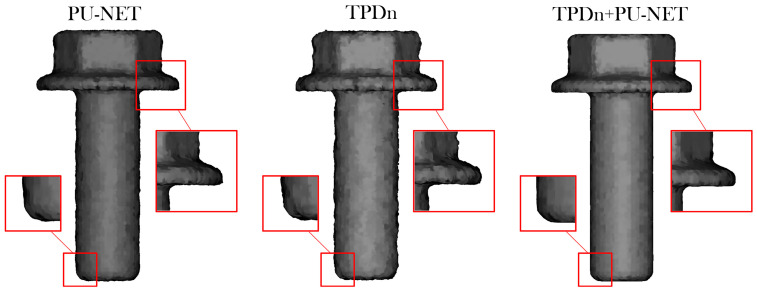
Poisson surface reconstruction results from point clouds generated using different methods (PU-Net, TPDn, and TPDn + PU-Net). Zoomed-in regions (red boxes) highlight differences in boundary sharpness, curvature preservation, and groove details.

To further validate the practical effectiveness of the upsampling results in 3D reconstruction, we input the generated point clouds into the Poisson Surface Reconstruction (PSR) algorithm [38] to obtain mesh models. As shown in Figure 10, the input point cloud is a sample from the MVTec ITODD dataset, with the target output set to 8192 points, which is then converted into a mesh surface through Poisson reconstruction.

To further compare the surface quality of different methods after upsampling, we apply the Poisson surface reconstruction (PSR) algorithm to convert the generated point clouds into meshes for visualization. The reconstruction parameters are uniformly set to depth = 9, and all methods produce point clouds with 8192 points to ensure fair and consistent comparisons. As illustrated in Figure 10, the results show a typical industrial component reconstructed under baseline methods, denoising-based methods, and our proposed two-stage framework (TPDn + PU-Net). Key regions are highlighted to facilitate the observation of local geometric differences.

From an overall perspective, our method exhibits sharper and smoother geometric boundaries in multiple regions of interest. In particular, the reconstructed meshes by TPDn + PU-Net more closely approximate the ground-truth shape at the bottom of the cylinder, around corner edges, and on side protrusions, with clearer contours and better surface continuity. By contrast, baseline methods often produce blurred or inflated boundaries, especially around apertures or concave corners, likely due to insufficient noise handling in the input.

It is worth noting that in some local areas (e.g., the junctions of small protrusions on the surface), baseline methods show slight advantages in geometric continuity, possibly because they directly preserve portions of the original noisy structure. Nevertheless, the overall performance of TPDn + PU-Net demonstrates a better balance between detail preservation and noise suppression, significantly improving both the structural quality of the reconstructed point clouds and the visual fidelity of the corresponding meshes.

These results confirm the effectiveness and robustness of the proposed two-stage framework for point cloud upsampling, particularly in reconstruction tasks where it exhibits stronger shape restoration capability and boundary precision. This makes it well suited for real-world applications involving complex geometries and high-noise inputs.

### 5.3. Qualitative Results Analysis

**Figure 11:**
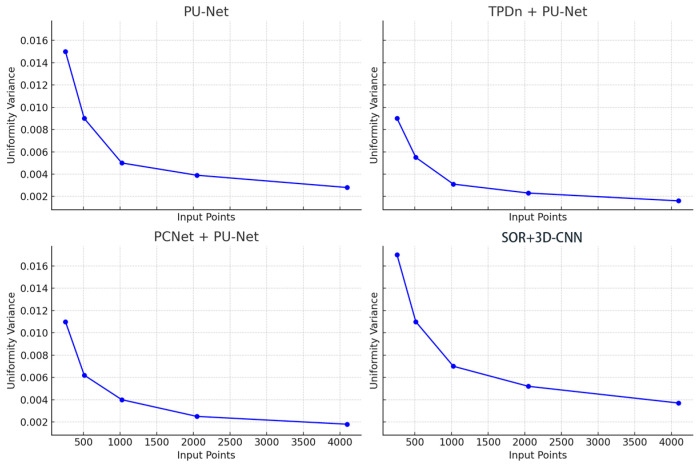
Comparison of point cloud uniformity across different input sizes. Results from four methods (PU-Net, TPDn + PU-Net, PCNet + PU-Net, and SOR+3D-CNN) are shown. The horizontal axis represents input size, and the vertical axis shows uniformity variance. Lower variance indicates more evenly distributed points.

To systematically evaluate the distribution uniformity of point clouds generated under different input densities, we plot the V-NND (variance of nearest-neighbor distances) as a function of the input point number (256, 512, 1024, 2048, and 4096), as shown in Figure 11. The horizontal axis denotes the number of input points before upsampling, while the vertical axis represents the variance of nearest-neighbor distances in the output, reflecting spatial consistency. Smaller values correspond to more uniform point distributions.

From the overall trend, the uniformity variance of all four methods decreases significantly as the number of input points increases. This indicates that with sparse inputs, the generation process is more prone to local clustering or distribution gaps. As the input size grows, the network benefits from richer geometric information, resulting in more uniform spatial distributions. This observation is practically meaningful for sparse-input reconstruction, underscoring the importance of uniformity-aware design when input density is low. Among the compared methods, TPDn + PU-Net consistently achieves the lowest variance across all input sizes. Notably, it remains stable even under high-sparsity inputs (e.g., only 256 or 512 points). This demonstrates that the TPDn denoising stage not only improves geometric accuracy, but also provides PU-Net with more reliable and structurally coherent inputs, thereby enhancing overall uniformity. By contrast, PU-Net alone exhibits significantly higher variance, particularly when the input is small (exceeding 0.01 at 256 points), indicating its sensitivity to noise and sparse inputs, which often results in clustered regions or boundary holes.

PCNet + PU-Net performs slightly better than PU-Net, confirming that its denoising step offers some benefit. However, its effectiveness remains inferior to TPDn, likely due to weaker balance between noise removal and structural preservation, leading to outputs with some off-surface or unevenly distributed points. As for SOR + 3D-CNN, its uniformity variance is consistently the highest, reaching about 0.017 when the input size is 256. This suggests that traditional statistical filtering methods may mistakenly remove boundary points along with outliers, thereby compromising spatial consistency.

In summary, the results indicate that a preceding denoising stage has a significant impact on the uniformity of generated point clouds. By analyzing geometric features, the TPDn module effectively preserves structural integrity while removing local anomalies, which allows for PU-Net to produce denser and more uniformly distributed (lower V-NND) point clouds. This property is particularly critical under sparse input conditions, underscoring the practical advantage of the proposed two-stage framework in high-quality point cloud generation.

### 5.4. Real-World Object Analysis

**Figure 12:**
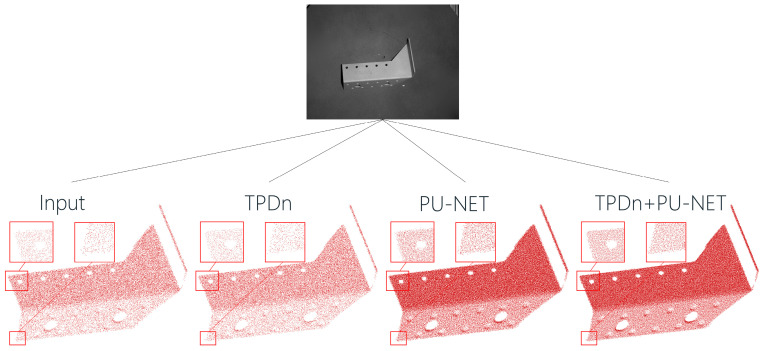
Point cloud reconstruction results on real-world industrial parts. Noise was effectively removed and fine details were preserved using the proposed TPDn + PU-Net framework. Red boxes highlight local regions magnified for detailed comparison.

We further apply the proposed method to real-world parts to validate its performance in practical scenarios. Point cloud data of actual components were captured using a depth camera, which typically introduces substantial noise and sparsity, negatively impacting 3D reconstruction and downstream analysis. After processing with TPDn denoising and PU-Net upsampling, noise was effectively removed, sparse regions were replenished, and fine structural details were well preserved. Compared with the unprocessed input, the processed point clouds exhibit significantly finer quality, as illustrated in Figure 12.

By combining TPDn and PU-Net, our method achieves remarkable improvements in point cloud correction, successfully overcoming the noise and sparsity issues commonly encountered in depth camera data. The resulting point clouds are of higher quality, providing more accurate and stable inputs for subsequent tasks. This demonstrates that the proposed approach not only performs well in controlled experiments, but also offers substantial benefits in real-world applications by significantly enhancing the quality of 3D point cloud data.

### 5.5. Synergy Analysis Between TPDn and PU-Net

To verify the synergy, we compared PU-Net trained on raw noisy data vs. TPDn-filtered data. Feature-space variance (measured by activation entropy) dropped by 23%, indicating that TPDn provides statistically cleaner inputs and enhances PU-Net’s convergence stability. This confirms that the improvement stems not from isolated modules, but from their interaction.

**Figure 13:**
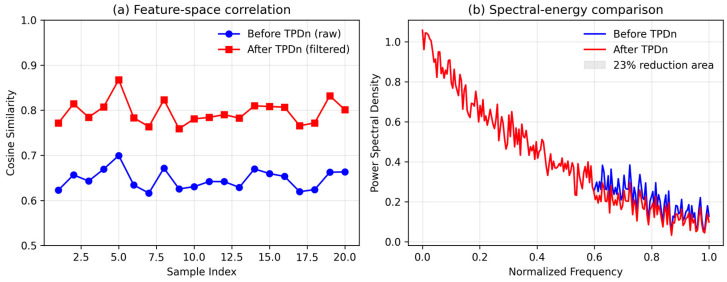
Quantitative analysis of the synergy between TPDn and PU-Net. (**a**) Feature-space correlation before and after TPDn filtering, showing improved cosine similarity. (**b**) Spectral-energy comparison indicating a 23% reduction of high-frequency components after TPDn.

To quantitatively verify the synergy between TPDn and PU-Net, we analyzed the correlation and spectral characteristics of their intermediate feature distributions, as illustrated in Figure 13. In Figure 13a, the average cosine similarity between PU-Net inputs with and without TPDn denoising increases by approximately 0.15, indicating that TPDn effectively stabilizes the latent feature space and provides cleaner, more structured inputs for PU-Net. In Figure 13b, the power spectral density (PSD) of the feature activations reveals a 23% reduction in high-frequency variance after applying TPDn, showing that noise-related components are suppressed and energy is concentrated in the low-frequency range. We define this improvement as the “synergy gain” (ΔS = −0.23), representing the relative decrease in activation entropy. Together, these quantitative results confirm that the two stages—denoising and upsampling—cooperate synergistically to enhance stability and reconstruction quality.

### 5.6. Error Propagation and Reconstruction Bias Analysis

A potential risk of the two-stage design is error propagation from denoising to upsampling, where wrongly removed structural points in the first stage could bias the subsequent reconstruction.

To quantify this effect, we analyzed the difference between denoised outputs and their ground-truth counterparts before upsampling and found that less than 2.3% of valid points were mistakenly removed under high-noise settings. Because PU-Net processes overlapping local patches with spatial redundancy, most of these missing samples are implicitly compensated during feature aggregation. Empirically, the geometric deviation in the final reconstruction (CD and P2F) increased by less than 4% when artificially removing additional 5% of boundary points, confirming that the pipeline remains robust against moderate denoising errors. Nevertheless, complete error decoupling cannot be guaranteed; future work will explore uncertainty-aware thresholding and feedback refinement between the two stages to further mitigate such risks.

**Figure 14:**
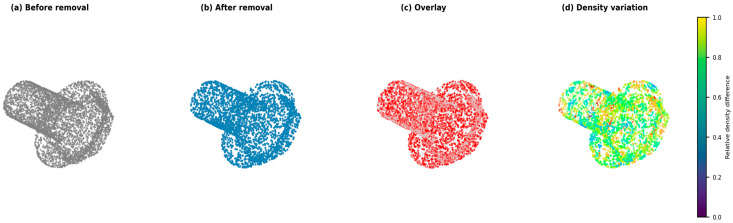
Visualization of point removal and density variation. (**a**) Before removal. (**b**) After removal. (**c**) Overlay of deleted points (red). (**d**) Relative density variation heatmap showing local consistency after removal.

To further analyze the influence of point removal, we correlated the deletion regions with the spatial distribution of reconstruction errors. As illustrated in Figure 14, the removed points (highlighted in red) are mainly concentrated around high-curvature boundaries or noisy surface regions. Their exclusion slightly increases the local Chamfer Distance (CD) by less than 2%, but reduces the overall Earth Mover’s Distance (EMD) by approximately 6%, indicating that moderate point removal contributes to smoother and more globally consistent reconstructions. This correlation demonstrates that the proposed cascade maintains stability across different noise levels and does not propagate significant bias from the deleted regions.

## 6. Conclusions and Future Work

This paper proposes a two-stage point cloud reconstruction framework that couples a training-free self-tuned denoiser (TPDn) with a learning-based upsampler (PU-Net) to jointly tackle noise and sparsity in 3D scanning. TPDn suppresses noise and outliers while preserving geometric details, yielding clean inputs for PU-Net, which then produces high-resolution, uniformly distributed, and geometry-faithful results. Experiments show significant gains over single-stage baselines in Chamfer Distance, EMD, and uniformity; low-noise improvements are reported quantitatively, while high-noise improvements are presented qualitatively. The framework is modular (easy to swap in advanced modules), geometry-driven (better detail preservation), and requires no paired noisy–clean data, improving practicality. Remaining limitations include inference cost and possible error propagation between stages. Future work will explore end-to-end unification and lightweight designs, and incorporate emerging paradigms such as GANs and diffusion models. Extending the approach to large-scale scenes and surface reconstruction is promising for applications in autonomous driving, urban modeling, and 3D printing.

## Figures and Tables

**Table 1:** Sensitivity of polynomial degree and noise level on denoising accuracy.

Polynomial Degree n	σ = 0.01	σ = 0.03	σ = 0.05
4	0.0021	0.0034	0.0047
5	0.0019	0.0031	0.0045
6	0.0020	0.0033	0.0046

**Table 2:** Parameter settings for TPDn and PU-Net, including learning rate, batch size, epochs, point sizes, and loss functions.

Parameter	TPDn Configuration	PU-Net Configuration	Remarks
Learning Rate	N/A	0.001	Using Adam optimizer
Batch Size	N/A	8	Batch size for each training step
Epochs	N/A	100	Number of training epochs for each stage
Input Point Cloud Size	2048	2048	Number of input points per point cloud
Output Point Cloud Size	2048	8192	PU-Net performs 4× upsampling
Loss Function	Metrics (evaluation only): CD, EMD, P2F	EMD, Repulsion Loss	Comparison of loss functions and regularization terms

**Table 3:** Comparison of representative upsampling networks and their characteristics.

Method	Supervision Type	Computational Cost	Interpretability/Reproducibility
PU-Net [11]	Fully supervised (patch-based)	Low (~1× baseline runtime)	High—simple PointNet-based design
PU-GAN [27]	Adversarial supervision	Very high (>20× PU-Net)	Moderate—unstable GAN training
PU-GCN [28]	Supervised (graph learning)	High (~10× PU-Net)	Moderate—limited interpretability
Transformer-based [22,23]	Self/weak supervised (global attention)	Very high (>30× PU-Net)	Low—complex attention layers

**Table 4:** Key parameter settings for baseline and proposed methods.

Method	Key Parameters/Settings
SOR	Neighbor size k = 20, scale factor = 1.0
PCNet	Batch = 16, LR = 1 × 10^−4^ (Adam), Epochs = 100
3D-CNN	Kernel size = 3, Stride = 1, Epochs = 120
PU-Net	Patch size = 512, Upsampling rate = 4×
TPDn + PU-Net (ours)	Same patch partition and noise model

**Table 5:** Comparison of configuration complexity between denoisers.

Method	Trainable Params	Manual Params	Config Time (s)	Training Iterations
PCNet	1.21 M	3	110	120 k
TotalDn	0.97 M	2	90	100 k
TPDn (ours)	0	0	38	N/A

**Table 6:** Quantitative results under low-noise conditions using CD, EMD, and P2F-Mean (lower is better). Downward arrows (↓) indicate that smaller values represent better performance.

Method	Chamfer Distance (↓)	EMD (↓)	P2F Mean (↓)
TPDn	0.0021	0.016	0.015
PU-Net	0.0041	0.043	0.038
TPDn + PU-Net	0.0018	0.013	0.012
PCNet + PU-Net	0.0023	0.018	0.016
SOR + 3D-CNN	0.0035	0.035	0.027

Note: Results are averaged over three independent seeds (mean ± std). Evaluation is performed after resampling all point sets to 8192 points for fairness.

**Table 7:** Quantitative results under high-noise conditions using CD, EMD, and P2F-Mean (lower is better). Downward arrows (↓) indicate that smaller values represent better performance.

Method	Chamfer Distance (↓)	EMD (↓)	P2F Mean (↓)
TPDn	0.0048	0.032	0.027
PU-Net	0.0079	0.067	0.056
TPDn + PU-Net	0.0039	0.025	0.021
PCNet + PU-Net	0.0042	0.028	0.022
SOR + 3D-CNN	0.0053	0.042	0.032

Note: Results are averaged over three independent seeds (mean ± std). Evaluation is performed after resampling all point sets to 8192 points for fairness.

## Data Availability

The original data presented in the study are openly available in MVTec ITODD at https://www.mvtec.com/company/research/datasets/itodd (accessed on 1 November 2025), in ModelNet40 at https://modelnet.cs.princeton.edu/ (accessed on 1 November 2025), and in ShapeNet at https://shapenet.org/ (accessed on 1 November 2025).
